# A pilot investigation of the prevalence of US-detectable forefoot joint pathology and reported foot-related disability in participants with systemic lupus erythematosus

**DOI:** 10.1186/s13047-016-0158-1

**Published:** 2016-08-02

**Authors:** Sandeep Mukherjee, Lindsey Cherry, Jalaa Zarroug, David Culliford, Catherine Bowen, Nigel Arden, Christopher Edwards

**Affiliations:** Faculty of Health Sciences, University of Southampton, Building 45, Burgess Road, Southampton, SO17 1BJ UK

**Keywords:** Lupus erythematosus, Systemic, Forefoot, Human, Joints, Bursa, Synovial, Ultrasonography

## Abstract

**Background:**

The main aim of this study was to determine the prevalence of US-detectable forefoot bursae, metatarsophalangeal (MTP) joint and metacarpophalangeal (MCP) joint synovial hypertrophy (SH), Power Doppler (PD) signal or erosion in participants with systemic lupus erythematosus (SLE). A secondary aim was to determine the strength of potential association between patient reported foot-related disability and US-detected forefoot bursae, MTP joint SH, PD signal or erosion in participants with SLE.

**Method:**

A cross-sectional observational study of 20 participants with SLE was completed to determine the prevalence of US-detected forefoot bursal, MTP and MCP joint pathology. Patient-reported foot-related impairment and activity limitation (accumulatively referred to as disability) were also recorded. Spearmans’ Rank Correlation analyses were completed to determine the potential strength of association between US-detected pathology and patient report disability.

**Results:**

The prevalence of MTP joint SH and PD was 80 % (16/20) and 10 % (2/20), respectively. The prevalence of MCP joint SH and PD was 60 % (12/20) and 30 % (6/20) respectively. A significant association was noted between PD scores for the MTP joints and MCP joints (*r* = 0.556; *p* = 0.011) although this was not demonstrated for SH scores (*r* = 0.176; *p* = 0.459). Significant associations between forefoot bursal prevalence and MTP joint PD were noted (*r* = 0.467; *p* = 0.038). The prevalence of bursae and bursal PD (grade 2 or above) was 100 % (20/20) and 10 % (2/20), respectively. Moderate foot-related impairment and activity limitation was reported by 95 and 85 % of participants respectively.

**Conclusion:**

This pilot study suggests that US-detected MTP, MCP joint and forefoot bursal abnormalities may be prevalent in participants with SLE and they may experience a moderate level of foot-related disability. Further research is required to substantiate these preliminary findings.

## Background

Systemic lupus erythematosus (SLE) is a chronic, autoimmune, inflammatory disorder of unknown aetiology that affects multiple organs including skin, soft tissues and joints [1]. In other similar inflammatory diseases like rheumatoid arthritis (RA), modern imaging techniques have demonstrated a high prevalence of foot problems that significantly reduce mobility and health-related quality of life [2]. However, the prevalence and impact of such problems in participants with SLE have not been well established to date and consequently there may be an unmet burden of poor foot health in this patient group despite previous evidence of metatarsophalangeal joint involvement [3].

Joint involvement in patients with SLE is common and it can have a significant impact upon the patients’ health-related quality of life [1]. This can reportedly vary from transient migratory arthralgia without objective evidence of synovitis to an erosive arthritis similar to RA [4]. There has been ongoing debate about whether this latter group represents an overlap of RA and SLE (rhupus) or a distinct subset of SLE [4, 5]. However, compared to other inflammatory arthropathies, joint disease in SLE has not been studied in much detail and the affectation of the metacarpophalangeal (MCP) joints or metatarsophalangeal (MTP) joints comparatively less well reported, particularly using US examination [4, 5].

The foot is a complex structure with numerous small anatomical details and differentiating between symptoms resulting from synovial hypertrophy, joint synovitis, tenosynovitis or bursitis can be problematic [2]. Clinical examination alone has been shown to be relatively insensitive to these features [6] and this is particularly relevant when the aim is to establish more targeted therapeutic approaches for patients with rheumatic conditions. Typically, MTP joint synovitis is associated with forefoot metatarsalgia and is thought to be due to the synovial lining becoming enlarged and inflamed as part of inflammatory disease [1, 2, 6]. Additionally, numerous small, synovially-lined bursae also exist throughout the forefoot and it is hypothesised that these may also become symptomatic as a consequence of poorly regulated inflammatory processes [2, 7]. However, it is unclear to date, to what extent patients with connective tissue disease, such as SLE, may also experience these pathological joint and tissue features.

Data from the FeeTURA programme of research suggests that US can readily differentiate between MTP joint hypertrophy, synovitis, bursal hypertrophy and bursitis as well as identify soft tissue pathology within the feet, which are not clinically apparent in patients with RA [2, 7, 8]. It is proposed that this technique may also be feasibly conducted in participants with SLE, although to our knowledge, this has not been trialed or reported to date. US has been widely used in RA to identify the extent of joint involvement as well as inform treatment decisions [9–11]. However, very few studies have used such assessments in participants with SLE to date [3]. Moreover, these studies have had relatively small numbers of participants and, apart from a recent study which also looked at abnormalities of MTP joints [3], they have mainly focussed upon wrist, hand or knee involvement [5, 12]. To our knowledge, this is therefore, the first study to explore the use of US for the investigation of MTP joint, MCP joint and forefoot bursal abnormalities in participants with SLE. The main aim of this study was to determine the prevalence of US-detectable forefoot bursae, MTP joint and MCP joint hypertrophy, Doppler signal or erosion in participants with SLE. A secondary aim was to determine the strength of potential association between patient reported foot-related disability and US-detected forefoot bursae, MTP joint hypertrophy, Doppler signal or erosion.

## Methods

### Study design

A cross-sectional, observational, study design was used for this project. The related study null hypotheses were defined as follows: (i) participants with SLE demonstrate no US-detectable pathology of the MTP joints or MCP joints and no reported foot-related disability, and (ii) there is no significant association between US-detectable forefoot pathology and reported disability in participants with SLE.

### Ethics, consent and permissions

Ethical approval for the study was obtained from the United Kingdom (UK) National Research Ethics Service (NRES) Committee North East Sunderland (13/NE/0161) and the study conducted in accordance with the Declaration of Helsinki guidelines for research (1975). All participants gave written informed consent and data was collected at the time of each participant’s scheduled rheumatology outpatient appointment.

### Participants

Individuals with a diagnosis of SLE confirmed by a consultant rheumatologist and fulfilling American College of Rheumatology (ACR) criteria [13] were consecutively and prospectively recruited from adult patients attending a UK rheumatology outpatient clinic. The 1997 criteria were pragmatically selected as these are used within the clinical environment in which the study was conducted [4]. As a novel pilot study, a sample size of 20 participants was pragmatically selected. Patients who had corticosteroid injection intramuscularly or to the forefoot or hand within the previous 3 months prior to study start, those with a diagnosis of concomitant musculoskeletal disease (e.g. primary osteoarthritis or gout), and those unable or unwilling to give consent were excluded from the study. All participants within this study had not received previous podiatry treatment or orthotic intervention.

### Assessment of disease activity

The Safety of Estrogens in Lupus Erythematosus National Assessment – Systemic Lupus Erythematosus Disease Activity Index (SELENA-SLEDAI) score was used to determine disease activity in participants with SLE [14]. This is a composite measure of organ and body system affectation as a consequence of SLE disease activity. An accumulative score is calculated following physician completion. The score range is from 0 to 105; where 0 indicates low disease activity and 105 indicates high disease activity. The presence of Rheumatoid factor, a blood borne immune marker, was also determined. The presence of this marker may indicate that a person could potentially have an overlap of disease between SLE and rheumatoid arthritis, which may lead to confounding when interpreting the findings of this study.

### US scan protocol

Grey scale (GS) and Power Doppler (PD) images were obtained using a diagnostic US scanner [Esaote MyLab70®]. All MTP joints and intermetatarsal spaces were imaged from dorsal and plantar approaches and in longitudinal and transverse planes using an 8-16 MHz linear array transducer. Simultaneous US assessment of MCP joints was conducted to compare disease activity in these joints with those of the MTP joints. All US scanning was performed in accordance with British Medical Ultrasound Society guidelines for safe use [15] and completed by an in-house trained researcher (SM). The reliability of this US protocol has been previously reported as moderate to substantial [8].

Joint synovial hypertrophy (SH) was identified as non-compressible hypoechoic intra-articular tissue on GS and graded between 0 and 3 (grade 0 = none; grade 1 = hypoechoic/anechoic line beneath joint capsule; grade 2 = elevation of joint capsule parallel to joint area, and grade 3 = bulge extending to at least one bone diaphysis either proximally or distally) [9–11]. The SH grades (0–3) for the 10 MCPJs or 10 MTPJs for each participants were combined to give an overall possible score range of 0–30 for the hands and feet respectively. PD activity was also graded semi-quantitatively as 0–3 (grade 0 = no flow; grade 1 = single-vessel signals; grade 2 = less than 50 % area of synovium filled with vessels and grade 3 = more than 50 % area of synovium filled with vessels) [10]. PD scores were combined as per SH scores for each participant to give an overall MCP joint or MTP joint score range of 0–30.

Intermetatarsal lesions were classified as hypertrophied bursae if a defined region of hypoechogenicity within intermetatarsal spaces, either inferior or superior to the deep transverse intermetatarsal ligament, was observed in transverse and longitudinal scanning planes, in a classification system consistent to that previously reported [2, 7, 8, 16]. Submetatarsal lesions were classified as hypertrophied bursae if a defined region of hypoechogenicity inferior to the base of metatarsal heads was observed similarly. For every participant, the presence or absence of bursa at each site was recorded giving a possible total score of 0–18 (5 submetatarsal and 4 intermetatarsal bursa in each foot) [8, 16]. Subsequently, the US grading (0–3) for PD signal obtained from each of the 18 bursal regions were combined to give a possible score range of 0–54.

Erosion was noted as present if a distinct loss in cortical bone was observed in two perpendicular scanning planes [10]. The total number of erosions identified for each participant was combined to give an overall MCP joint or MTP joint score range of 0–10.

### Assessment of patient-reported disability

Patient-reported foot-related impairment and activity limitation was assessed using the ‘Foot Impact Scale’ (FIS) questionnaire [17]. This questionnaire has two subscales: ‘foot impairment and footwear restriction’ (FIS_IF_, score range 0–21) and ‘activity limitation and participation restriction’ (FIS_AP_, score range 0–29), and it has previously been validated for use in patients with rheumatoid arthritis [17]. An elevated FIS_IF_ or FIS_AP_ score indicates greater foot impairment or activity limitation, respectively. Henceforth, the FIS_IF_ subscale will be referred to as ‘foot impairment’ and the FIS_AP_ subscale as ‘activity limitation’. The FIS questionnaire was selected because, to our knowledge, this is the only tool with foot related subscales that differentiate disability from pain. We are not aware of any currently validated tools for the evaluation of patient-reported foot-related disability in participants with SLE.

### Analysis

All analysis was completed using Stata version 11.0 (Stata Corp, USA), or SPSS version 18.0 (Chicago, USA). Prior to analysis, data was checked for inconsistencies, outliers, missing information and distribution. Participant characteristic data measured using a continuous scale are presented as means and standard deviations (SDs) if approximately normally distributed, and as medians and inter-quartile ranges (IQRs) if skewed. Statistical significance was reported at 5 % (i.e. *p* < 0.05) confidence level, based upon two-tailed analysis.

All variables undergoing correlation analysis were identified as negatively skewed, and as such, a non-parametric test of association (Spearman’s Rank Correlation Coefficient) was used. Tests of association were explored between hand and foot data, and US variables and patient-reported foot impairment or activity limitation.

## Results

The mean (SD) age of participants was 53.6 (12.8) years. There were 18 females and 2 males. Mean (SD) disease duration was 12.1 (7.6) years and overall disease activity was low with the median SELENA-SLEDAI score for the cohort being 0 (IQR: 0 to 1). All participants were in receipt of disease modifying agents and 55 % (11) were receiving oral steroids with median daily Prednisolone dose of 10 mg (IQR: 5 to 15 mg). Rheumatoid factor (RF) was negative in eleven (55 %) and positive in five (25 %) of the participants. RF status was not known for the remaining four participants.

The US findings have been summarised in Table 1. The prevalence of MTP joint SH and PD was 80 % (16/20) and 10 % (2/20), respectively. The prevalence of MCP joint SH and PD was 60 % (12/20) and 30 % (6/20), respectively.

**Table 1:** Summary findings of musculoskeletal US scanning of the forefeet and hands

	Prevalence % (*n*)	Median Score	Interquartile range
MCP Joint SH	60 (12)	1.5	0–3.75^b^
MCP Joint PD	30 (6)	0.0	0–1^b^
MTP Joint SH	80 (16)	2.0	1–2^b^
MTP Joint PD	10 (2)	0.0	0–0^b^
Bursa^a^ present	100 (20)	9.5	8–11.75^c^
Bursa^a^ PD	100 (20)	3.0	2–3.75^d^

^a^Submetatarsal and intermetatarsal bursa

^b^Out of a possible combined score range of 0–30(using US grading of 0 to 3 for both SH and PD for each of the 10 MCP and10 MTP joints)

^c^Out of a possible score range of 0–18 (5 submetatarsal and 4 intermetatarsal bursa on each foot)

^d^Out of a possible combined score range of 0–54 (using US grading of 0 to 3 for each of the 18 bursal spaces imaged per patient)

Overall, a number of participants demonstrated SH in at least one MTP or MCP joint. PD grade 1 or above in at least one MTP or MCP joint was seen in two and six participants, respectively. One participant had PD grade 2 observed in an MCP joint. All participants demonstrated at least one hypertrophied bursa with PD grade 1 signal. Two participants had a single bursa with PD grade 2 or above. Thus, the first null hypothesis can be rejected.

Correlation analysis determined that those participants with SH in their MTP joints did not necessarily also have it in their MCP joints (*r* = 0.176; *p* = 0.459). However, a significant association was noted between PD scores for the MTP joints and MCP joints (*r* = 0.556; *p* = 0.011).

A significant associations between forefoot bursal prevalence and MTP joint PD was noted (*r* = 0.467; *p* = 0.038), but this was not observed between bursal prevalence and MCP joint PD (*r* = 0.577; *p* = 0.133). Additionally, a significant association between bursal PD and MTP joint PD was noted (*r* = 0.460; *p* = 0.041).

Characteristics of participants and their respective foot impairment and activity limitation scores have been summarised in Table 2. Foot-related impairment or activity limitation was reported by 95 and 85 % of participants, respectively. The median score for foot impairment was 11 (IQR: 4.8 to 14.0) and activity limitation was 11.5 (IQR: 1.0 to 20.5).

**Table 2:** Demographic characteristics of 20 SLE participants and their self-reported foot impairment and activity limitation

Participants	Age (years)	Sex	Duration of SLE (years)	RF positivity	FIS_IF_	FIS_AP_
1	46	Female	9	Negative	12	1
2	51	Female	5	Positive	8	10
3	66	Female	36	Negative	13	1
4	56	Female	18	Negative	10	19
5	40	Female	14	-	1	0
6	55	Female	19	Positive	9	2
7	62	Female	11	Negative	15	21
8	41	Female	2	-	4	0
9	60	Female	13	Positive	18	17
10	63	Female	15	Positive	0	0
11	57	Female	9	Negative	16	29
12	45	Female	12	-	9	11
13	60	Male	8	Negative	7	7
14	63	Male	7	Positive	13	16
15	38	Female	14	Negative	14	21
16	73	Female	7	-	14	27
17	64	Female	5	Negative	13	11
18	67	Female	22	Negative	4	8
19	44	Female	8	Negative	3	1
20	20	Female	7	Negative	16	22

*FIS*_*IF*_ foot impairment and footwear restriction

*FIS*_*AP*_ activity limitation and participation restriction

Patient-reported foot impairment and activity limitation were not significantly associated with MTP joint SH (*r* = −0.044; *p* = 0.852 and *r* = −0.170; *p* = 0.474, respectively), MTP joint PD (*r* = 0.333; *p* = 0.151 and *r* = 0.421; *p* = 0.065, respectively), prevalence of bursa (*r* = 0.237; *p* = 0.314 and *r* = 0.186; *p* = 0.433, respectively), or bursal PD (*r* = 0.274; *p* = 0.243 and *r* = 0.379; *p* = 0.099, respectively). Thus, the second null hypothesis can be accepted.

## Discussion

This pilot study suggests that US detected synovial and bursal abnormalities within the MTP and MCP joints of participants with SLE are prevalent and hence, could be potentially under-recognised clinically. A significant association between US-detectable MTP joint Doppler activity and bursa was noted. Additionally, participants reported moderate levels of foot-related impairment and activity limitation, respectively, which could be considered comparable to that reported by patients with RA [7]. However, unlike previous work in participants with RA, no association between US-detected joint pathology and patient reported disability was observed in this study [7]. It is currently not possible to comparatively evaluate the prevalence of US-detected pathology found in this study as to our knowledge, this is the first pilot study to do so.

The prevalence of US detected MTP and MCP joint and forefoot bursal abnormalities in a cohort of participants with relatively low systemic markers of SLE activity shown in this study suggests that there may be a persistent level of disease activity that is clinically under-recognised. Conversely, the presence of joint hypertrophy or bursal lesions may be related not to inflammatory disease but mechanical tissue irritation. Further work is required to fully appreciate the clinical interpretation of increased forefoot bursae in this participant group. Similarly, the moderate levels of patient reported foot-related impairment and activity limitation may represent a further independent or related unmet foot health burden. Nonetheless, this pilot data suggests that there may be a need for further US examination to fully appreciate disease activity and prevalence in this patient group. However, the aetiology and/or risk factors leading to these findings remain unclear.

Although joint and bursal abnormalities within the forefeet were apparent in this pilot study, they do not appear to be significantly associated with reported foot-related impairment or activity limitation. However, the sample size for this study is small and as such association analyses of this kind are potentially subject to related bias and error. Nonetheless, the findings suggest the need for further research to fully appreciate the potential burden of poor foot health in patients with SLE, the associated risk factors and thus potential therapeutic targets.

This study has several potential limitations. Firstly, as a pilot study, a small number of participants were included. Further studies with larger numbers of participants are needed to substantiate any trends shown here. Secondly, the cohort of participants with SLE studied generally had mild disease and fuller data regarding disease status or ethnicity is required. Future studies could investigate participants across the spectrum of disease severity, including those at the severe end. Moreover, anti-CCP (anti-cyclic citrullinated peptide) antibody status for all participants could be determined and those testing positive excluded from the study to rule out possibility of any overlap with RA [1, 4, 13]. In addition, evaluation of potential confounding factors (e.g. presence of concomitant disease) would be feasible with replication of this protocol in a larger cohort, and the data presented here may be used to inform such sample size calculations. Thirdly, the 2012 ACR classification criteria were not used and the 1997 criteria were pragmatically selected as these are used within the clinical environment in which the study was conducted [4]. Finally, there are no current guidelines as to the minimum standards of sonography training in order to undertake and use US within clinical or research practice, especially with regards to standardised approaches in participants with SLE [9, 15, 18]. The physician undertaking the US examination as part of this study (SM) has received substantial ‘in-house’ training, which has previously been shown to have good intra-rater agreement, however it should be acknowledged that he is not a full-time Sonographer [8]. There is a need for standardisation of US protocols of the foot and ankle (including probe selection and machine settings) and training requirements to help ensure consistency and accuracy in approach. For example, arguably, a higher frequency probe may allow improved visualisation of superficial structures, whilst a lower frequency probe would do the converse; thus appropriate probe selection and availability may influence image acquisition and interpretation accuracy [3, 9]. Standardisation of such protocol elements will further assure clinicians as to the accuracy of research that is conducted using this technique.

## Conclusion

In this novel work, participants with SLE were shown to have a high prevalence of US-detectable MTP and MCP joint SH, PD and bursal abnormalities. Additionally, this patient group reported moderate levels of foot-related impairment and activity limitation. The data presented can be used to inform the basis for future larger cohort investigations with potentially high clinical relevance to the ongoing foot health of patients with SLE.

## Abbreviations

ACR: American college of rheumatology; Anti-CCP: anti-cyclic citrullinated peptide; FeeTURA: foot and ankle ultrasound studies in rheumatoid arthritis; FIS: foot impact scale; FIS_AP_: activity limitation and participation restriction; FIS_IF_: foot impairment and footwear restriction; GS: Grey scale; IQR: inter-quartile range; MCP: metacarpophalangeal; MTP: metatarsophalangeal; NRES: national research ethics service; PD: power doppler; RA: rheumatoid arthritis; RF: rheumatoid factor; SD: standard deviation; SELENA-SLEDAI: safety of estrogens in lupus erythematosus national assessment - systemic lupus erythematosus disease activity index; SH: synovial hypertrophy; SLE: systemic lupus erythematosus; US: Ultrasound
